# Sensitive Detection of Dendritic Lithium Morphologies by Dynamic Nuclear Polarization

**DOI:** 10.1021/acs.jpclett.5c02140

**Published:** 2025-08-20

**Authors:** Nadav Maimon, Ayan Maity, Xiao-Meng Sui, Michal Leskes

**Affiliations:** † Department of Molecular Chemistry and Materials Science, 34976 Weizmann Institute of Science, Rehovot 761000, Israel; ‡ Department of Chemical Research Support, 34976 Weizmann Institute of Science, Rehovot 761000, Israel

## Abstract

Lithium metal batteries are a promising energy storage technology, but their commercialization is hindered by nonuniform lithium deposition, which is detrimental to the battery lifetime and safety. In particular, needle-like dendrites pose the greatest risk as they often lead to short-circuits; as such, it is essential to identify and mitigate their formation for enabling use of lithium metal anodes. Here we demonstrate that Overhauser dynamic nuclear polarization (DNP)- enhanced NMR, where the high polarization of the lithium conduction electrons increases the sensitivity of lithium NMR, is a powerful tool for determining the lithium morphology. By systematically controlling the deposited lithium structures within a polymer electrolyte system, we show that DNP enhancement correlates with morphology, allowing us to distinguish between micro- and nano-sized dendrites. Complementary electron paramagnetic resonance and electron microscopy measurements confirm the morphological interpretation. This work introduces a spectroscopic strategy for sensitively probing lithium dendritic structures with high specificity, offering a pathway to understand and control their formation across a range of battery systems and electrochemical formation conditions.

## Graphical abstract

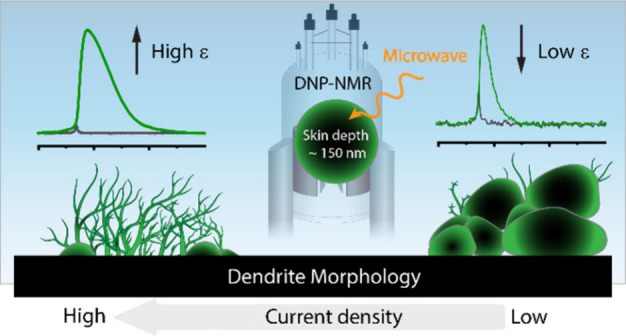

Lithium-metal batteries (LMBs) offer a promising path to overcome the energy density limitations of current lithium-ion batteries, enabling their use in high-energy applications.^1–3^ However, lithium metal reactivity and the associated safety concerns currently prevent LMB commercialization. Specifically, the main challenge with the utilization of lithium metal is its continuous reactivity with the electrolyte. Ideally, electrolyte degradation should occur only in the first few cycles, resulting in deposition of solid phases at the electrode–electrolyte interface and formation of the solid–electrolyte interphase (SEI).^4^ A beneficial SEI would passivate the electrode surface and allow efficient and homogeneous ion transport across it. In practice, the formation of nonuniform interphases results in “hot spots” for lithium deposition which then lead to nonuniform lithium plating and growth of lithium nano- and microstructures.^5–8^ Such high surface area lithium deposits lead to further consumption of the electrolyte, but even more critically, if the deposits grow in needle-like structures which are often referred to as dendrites, they can short-circuit the battery cell. As such, these dendritic structures prevent the use of lithium metal with common liquid electrolytes as they tend to catch fire when the cell is short-circuited. Thus, there is an urgent need to identify the conditions that lead to formation of dendritic lithium deposition vs the less hazardous smooth and mossy deposition. The morphology of lithium deposits is a result of the complex interplay between electrolyte and SEI ionic conductivity and surface diffusion of atomic lithium.^9^ Furthermore, it depends on many factors in the battery cell including current density, electrolyte composition and properties, cell pressure and temperature.^6^ In order to understand how these different factors affect deposition, we must be able to distinguish between the different morphologies formed under different cycling conditions and, in particular, identify the initiation of dendritic formation.

To date, several approaches have been employed to investigate lithium morphology. These include electron microscopy (EM)^10–13^ and computed tomography (CT).^14–16^ The difficulty in utilizing EM is that it does not provide full quantitative insight: this would require multiple image collections and analyses. Furthermore, the lithium is imaged along with the SEI layer on top of it, which makes it difficult to assess the metallic domain sizes. CT provides better overview of the morphologies but again relies on sufficient signal collection and does not always provide the needed resolution. Magnetic resonance-based techniques are advantageous as they provide a quantitative, nondestructive approach that is sensitive to the entire sample composition. Electron paramagnetic resonance (EPR) is a powerful approach to determine the size of the metallic lithium microstructures^17–21^ in a quantitative manner, but when the sample contains several morphologies, it is difficult (and often impossible) to identify the contribution of dendritic structures in the presence of micron-size morphologies.^6,7^ Li solid-state nuclear magnetic resonance (ssNMR) spectroscopy has better chemical resolution than EPR, yet it has much lower sensitivity and in the presence of both mossy and dendritic structures it also lacks selectivity.^22–25^ Dynamic nuclear polarization (DNP)^26,27^ offers a path to overcome these challenges as it combines the benefits of both methods. By taking advantage of the electrons’ high spin polarization, lithium metal conduction electrons are used to hyperpolarize the lithium nuclei.^28,29^ Thus, DNP enhances the NMR signal, allowing sensitive characterization of lithium nano-/microstructures as well as the composition and properties of the SEI formed on them.^20,30–32^

Here we show that in addition to sensitivity DNP provides an effective route to distinguish the different lithium deposition morphologies, with high selectivity toward dendritic nanoscale structures. We demonstrate this approach by investigating lithium morphologies formed within polymer electrolytes. Polymer electrolytes offer a safer path for utilization of LMB;^33–35^ however, they are far less explored in terms of their susceptibility to dendrites formation.^6^ By systematically varying the current density in symmetric Li-metal cells assembled with a polymer electrolyte, we induced the formation of lithium deposits with distinct morphologies. Detailed DNP-NMR characterization revealed that the different morphologies exhibited markedly different enhancement factors and spectral features. We support these findings with EPR measurements, which show clear differences in spectral properties in correlation with dendrite morphology. Furthermore, EM analysis is provided to complement insights into the deposits’ size distribution and surface roughness. Finally, we discuss the differences between the approaches, highlighting the selectivity gained by DNP-NMR which provides a comprehensive framework for understanding the structure–property relationship of Li dendrites.

Symmetric lithium cells were prepared separated by a poly­(ethylene oxide) (PEO) membrane containing lithium bis­(trifluoromethanesulfonyl)­imide (LiTFSI) salt. This system was chosen as it does not pose a safety concern and from the methodology point of view, we have shown that it provides an efficient framework to study dendrites by DNP-NMR.^31^ An additional advantage of using this polymeric medium is its ability to encapsulate the lithium structures during cycling, which protects their native morphology. Post-cycling, the polymer can be easily extracted, allowing the embedded metallic lithium to be directly analyzed by NMR and other complementary characterization techniques. Figure 1a shows the PEO electrolyte post-cycling, after cell disassembly. It is established that in liquid electrolytes the current density, i.e., the rate of lithium plating and stripping, has critical influence on lithium deposition morphology.^5,6^ Following this approach, we assume that current density will also affect deposition in polymer electrolytes. The battery cells were cycled with polymer electrolytes under two distinct current densities: 0.025 and 0.5 mA/cm^2^. Figure 1b,c shows the lithium stripping and plating profiles of the symmetric cells under the two current conditions. The profiles are clearly distinct: at the lower current density, the stripping and plating cycles lasted 1 h (the cutoff time given) with relatively low overpotentials. In contrast, at the higher current density, the cycling duration was much shorter due to the rapid rise in overpotential, which quickly reached the cutoff voltage. These cells also short circuited quickly unlike cells cycled with low current density. Following cycling, the batteries were disassembled and the cycled polymer sandwiched between the two lithium electrodes was extracted and used for micro-CT studies. Micro-CT analysis of the sample cycled at 0.5 mA/cm^2^ (Figure 1d) clearly reveals the presence of lithium microstructures that penetrate the polymer matrix. A full 3D reconstruction of the cross-section is provided in the Supporting Information (Figure S1 and video file). While visually informative, the spatial resolution of CT is not sufficient for extracting precise morphological features.

**Figure 1:**
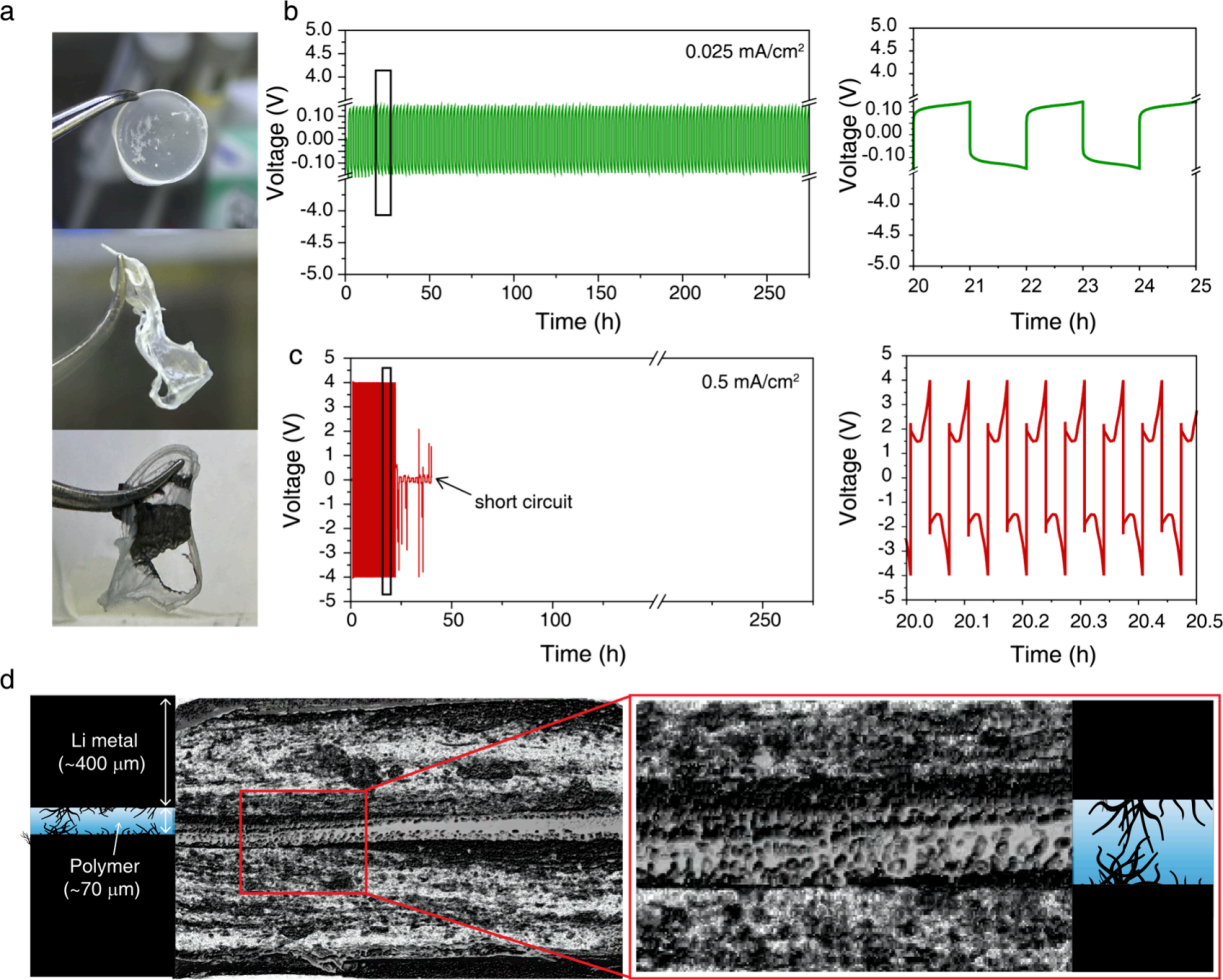
(a) Optical images of the polymer electrolyte in three states: pristine (top), cycled at 0.025 (middle), and cycled at 0.5 mA/cm^2^ (bottom). The appearance of gray spots in the cycled samples indicates metallic lithium embedded within the polymer matrix. (b, c) Voltage vs time profiles of symmetric Li-metal cells cycled at 0.025 and 0.5 mA/cm^2^, respectively. (d) Micro-CT cross-sectional images taken from a cell cycled at 0.5 mA/cm^2^, where the zoomed-in region (red box enlarged in the bottom image) reveals significant dendritic growth. Schematic representations depict the interpretation of the observed features.

To investigate and characterize the morphologies formed under different current density conditions, the polymer that was now cycled between the ^6^Li-rich electrodes was extracted from the battery and directly packed into the sealed DNP-NMR rotor for solid-state NMR experiments. We utilized ^6^Li enriched samples to improve the NMR spectral resolution as well as benefit from the longer relaxation times of ^6^Li. This approach was chosen to mitigate the broadening and fast relaxation typically observed with ^7^Li, which arise from its strong dipolar interactions and quadrupolar nature (spin 3/2). In contrast, ^6^Li (spin 1) has a lower quadrupole moment and gyromagnetic ratio. Both lead to significantly narrower lines, enhanced spectral resolution, and longer relaxation times, which lead to higher DNP enhancements. In the ssNMR spectrum, the Li-metal signal typically appears around 260 ppm due to the Knight shift, while diamagnetic components such as SEI and the electrolyte resonate near 0 ppm. Under microwave irradiation and with sufficient microwave power for Overhauser DNP, signal enhancement is achieved by saturating (at least partially) the EPR transition of the conduction electrons in the metal. The resulting nuclear polarization enhancement occurs via a cross-relaxation mechanism, which is driven by the spatial fluctuations of the conduction electrons in the metal. This mechanism enables significant enhancement of the Li-metal signal, providing a sensitive means to probe the metal deposits nondestructively. The efficiency of this process is governed by the microwave skin depth, which defines how deep the microwaves penetrate the metal. For lithium metal at 100 K on a 9.4 T magnet with a resonance frequency of 263 GHz, the skin depth is approximately 130 nm (see Supporting Information). When the metal structures are smaller than this depth, full penetration enables maximal DNP enhancement, as all the nuclei in the dendrites are contributing to the enhanced signal. Larger structures, however, exhibit attenuated enhancement due to limited microwave penetration compared to the larger thickness detected by NMR (with radiofrequency excitation). Thus, our expectation is that this size dependence makes the DNP enhancement factor a useful proxy for probing metallic lithium morphologies. Specifically, we expect that nanosized dendrites, with high surface-to-volume ratio and small grain size, will produce significantly higher enhancement than bulkier, mossy dendrites.

Figure 2a,b shows the ^6^Li DNP-NMR spectra acquired with (ON) and without (OFF) microwaves at 100 K for samples cycled at 0.025 and 0.5 mA/cm^2^, respectively. The average metallic lithium enhancement factor increases from ∼14 at 0.025 mA/cm^2^ to ∼78 at 0.5 mA/cm^2^ (Figure 2c). These results support that higher current densities promote dendritic structures formation with smaller metallic domain sizes and higher surface areas, most likely needle-like dendrites, compared to the bulkier mossy morphology formed at low currents. An intermediate current density (0.125 mA/cm^2^; Figure S2) yields an enhancement factor consistent with a mixed population of both morphologies. With microwave irradiation, the line width and shape of the metallic resonance change due to a reduction in the Knight shift, which originates from Fermi contact interactions between nuclear spins and conduction electrons. It can be monitored by the full width at half-maximum (fwhm). When the EPR transition is saturated, the electron spin polarization decreases, decreasing the Fermi contact interaction and resulting in a reduction of the Knight shift. As EPR saturation becomes more effective, the Knight shift further decreases. As this effect is not uniform across the heterogeneous (in terms of structural defects, electric conductivity, and electron spin magnetic resonance properties) lithium deposits, it results in peak broadening. Therefore, nanoscale dendrites are expected to have a larger fwhm compared to micron-size structures. A significant increase in fwhm is observed upon increasing the current density: from ∼9 ppm at 0.025 mA/cm^2^ to ∼27.5 ppm at 0.5 mA/cm^2^ (Figure S3a). This broadening is consistent with a reduction in dendrite grain size and overall morphology, with a length scale closer to the skin depth. As a result, microwave penetration becomes more efficient and, therefore, also the EPR saturation. Consequently, the observed fwhm increase serves as an indirect indicator of smaller, higher surface area morphologies. Additionally, morphological heterogeneity and variations in dendrite shape and size contribute to further peak broadening. However, this effect is likely secondary, as larger lithium size distribution and structural heterogeneity are observed at a lower current density (see below), and are associated with narrower peaks.

**Figure 2:**
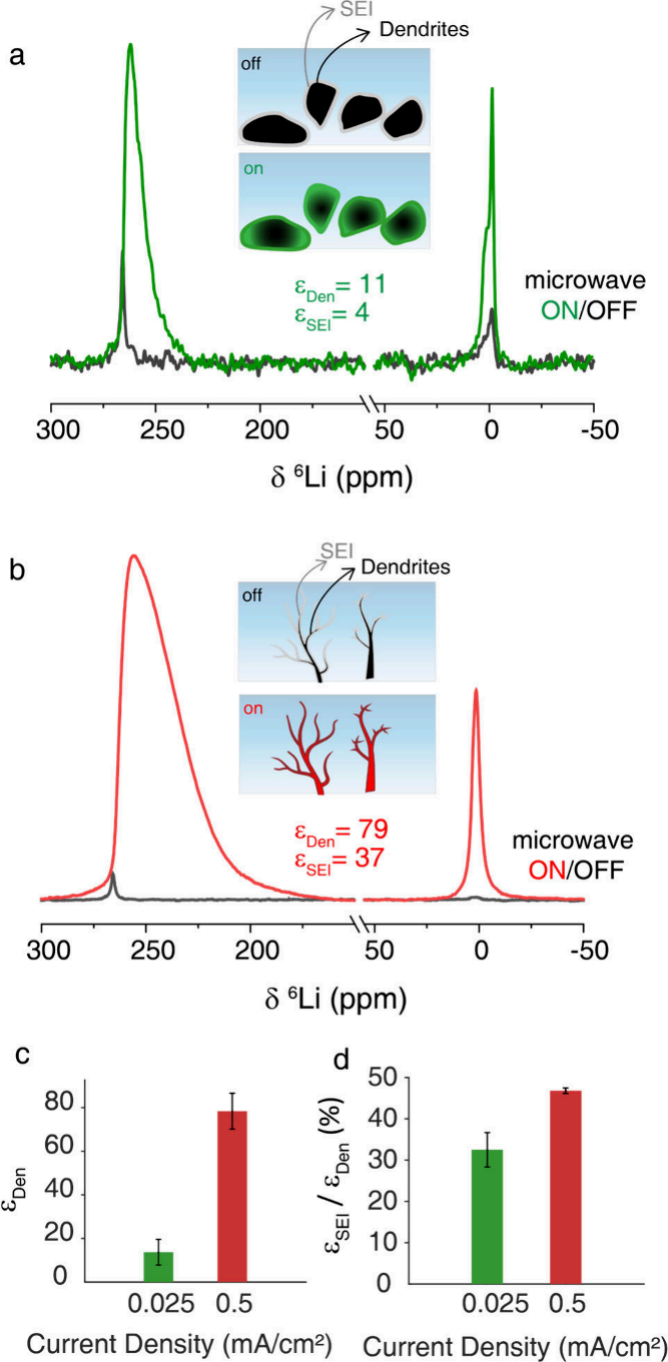
^6^Li DNP-NMR microwave on/off spectra of PEO samples cycled at (a) 0.025 and (b) 0.5 mA/cm^2^. Inset: Schematic representation of the microwave-off and on conditions highlights that the morphology of the lithium deposits affects DNP efficacy. (c) Metallic lithium enhancement and (d) SEI/dendrite enhancement ratio as a function of current density. DNP experiments were performed at 100 K with a 2000 s recycle delay and 8 kHz magic angle spinning (MAS). Error bars represent the standard deviations obtained from measurements of three independent samples.

The SEI signal is also enhanced through charge transfer with the metal, as previously shown by Maity et al.^31^ and validated in our system (Figure S3b). Figure 2d shows the SEI/dendrite enhancement ratio as a function of current density. This ratio provides insight into the fraction of the SEI that is in direct contact with the metal surface; a higher ratio results in a higher lithium charge transfer rate. Our results reveal an increase in the SEI/metal enhancement ratio from ∼32.5% at 0.025 mA/cm^2^ to ∼46.8% at 0.5 mA/cm^2^, which can be an indication of greater interfacial contact at higher current densities. Importantly, the enhancement of the SEI resonances now enables chemical analysis of the SEI composition, which would otherwise be impossible. As shown in Figure S4, the SEI formed at both current densities is primarily composed of LiOH and Li_2_O. In several samples cycled at 0.5 mA/cm^2^, an additional peak (possibly Li_3_N) at ∼8 ppm is observed.

To support our morphological interpretation, we have also employed the more common analysis tools: scanning electron microscopy (SEM) and EPR. As discussed, the DNP efficiency is directly influenced by the extent of the saturation of the EPR transition. These are, in turn, affected by the microwave skin depth and the lithium morphology. EPR is particularly well-suited for metallic lithium characterization due to the low spin–orbit coupling of lithium, which yields a sharp resonance with line shape that is directly related to the metal morphology. Throughout this work, EPR data are shown as first-derivative line shapes. Two parameters are commonly used to analyze the EPR line shape: the A/B ratio and the fwhm.^18^ The A/B ratio is defined as the amplitude of the EPR signal maximum (A) relative to the minimum (B), while the fwhm corresponds to the field difference between these points. Both parameters are influenced by the microwave skin depth (∼1.6 μm for lithium at X-band 0.3 T and 9.4 GHz where the EPR measurements were performed, see Supporting Information). When the lithium structures are smaller than the skin depth, the microwave field uniformly penetrates the sample, producing a symmetric line shape with A/B ≈ 1. In contrast, larger structures experience partial field penetration, resulting in signal asymmetry (A/B > 1) and broader fwhm values.^36^ Additionally, power saturation behavior reveals information about electron spin relaxation: finer structures saturate at lower microwave powers, while bulkier structures require higher power or may not saturate at all.^37^ Figure 3a presents a typical EPR spectrum of the lithium trapped in PEO formed under 0.5 mA/cm^2^ cycling, measured at room temperature with 5 mW microwave power. The spectrum displays the expected resonance shape, with marked A and B points and fwhm. Figure 3b shows the power saturation behavior—peak-to-peak amplitude vs squared microwave power—for lithium deposition at both high and low current densities. Lithium structures grown at 0.5 mA/cm^2^ exhibit signal saturation at lower power compared to those formed at 0.025 mA/cm^2^ which do not reach saturation within the measured power range. For reference, bulk lithium shows a linear unsaturated response. Figure 3c,d shows the A/B ratio and fwhm, respectively, as a function of the squared microwave power. Across the full power range, samples cycled at 0.5 mA/cm^2^ consistently exhibit A/B ratios close to 1 and narrow peaks, in contrast to structures formed at 0.025 mA/cm^2^ with higher A/B ratios and broader peaks. For comparison, bulk lithium metal (Figure S5) exhibits a much broader and less symmetrical EPR signal. Both the A/B ratio and fwhm remain relatively constant at low saturation power and increase in magnitude at higher powers, reflecting the progressive excitation of previously inaccessible, more bulk-like domains (see discussion below).

**Figure 3:**
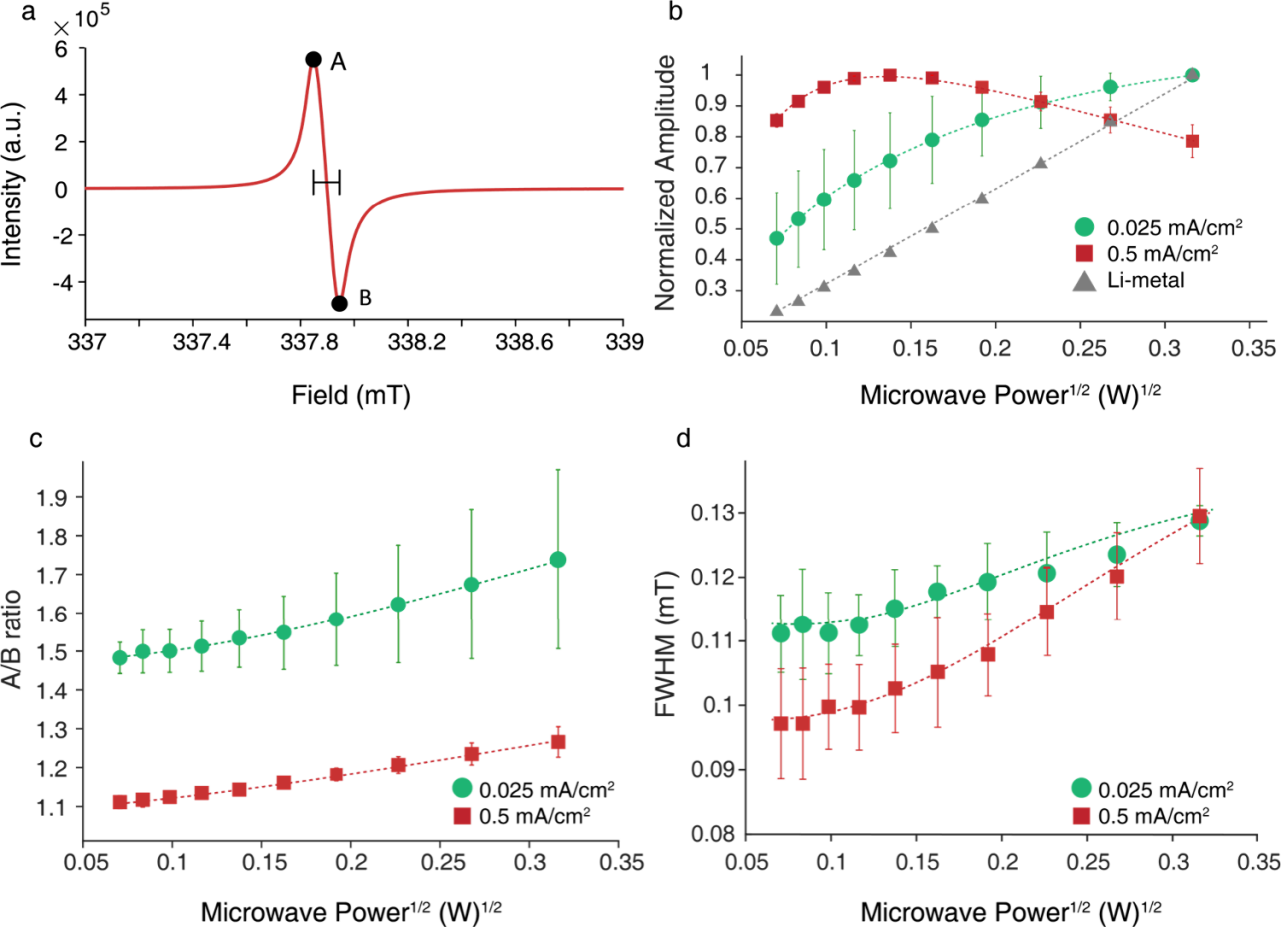
(a) EPR line shape of samples cycled at 0.5 mA/cm^2^ measured with 5 mW microwave power. (b) Normalized peak-to-peak amplitude of EPR line vs squared microwave power for samples cycled at 0.025 (green) and 0.5 mA/cm^2^ (red), and uncycled bulk lithium metal (gray). A/B ratio (c) and fwhm (d) of EPR line vs squared microwave power for samples cycled with 0.025 (green) and 0.5 mA/cm^2^ (red). All the measurements were done on a benchtop X-band system at 298 K. Error bars represent the standard deviation obtained from measurements of three independent samples.

All three measurements—power saturation curves, A/B ratio, and fwhm—strongly support the formation of dendritic, thin lithium structures at high current densities with larger morphologies formed at lower current densities. In particular, the symmetric line shape observed for lithium deposits at high current densities is a very strong indication that the metallic domains formed are smaller than the skin depth of 1.6 μm at the measurement field. At low current densities, the lithium deposits are larger than this skin depth, yet they are still far in size from bulk lithium, as indicated by measurements performed on a chunk of lithium metal. It is common to associate the fwhm of metallic resonances with electron spin relaxation, with narrower lines corresponding to longer relaxation times. Here we observe that dendritic deposits have significantly longer electron relaxation times. This is also consistent with the power saturation curves, with increasing saturation efficiency, indicating longer electron relaxation times. The domain size of metallic particles has two opposing effects on electron relaxation: On one hand, as the particles get smaller, the contribution from surface scattering events can lead to shortening of electron relaxation times. On the other hand, for domains in the nanoscale it was shown that electron relaxation times become longer due to quantum size effects.^38^ Here we observe that the dendritic structures result in longer relaxation times, which is likely another major factor leading to their higher enhancement in OE-DNP. Based on this observation, with higher saturation power we expect increasing contribution from lithium particles with shorter relaxation times. Here shorter relaxation times are associated with a larger A/B ratio, suggesting they have a larger grain size. Together, these EPR results strongly corroborate the DNP findings; higher current densities promote the growth of smaller, nanoscale dendrites with higher surface area, while lower current densities yield larger and more heterogeneous morphologies. However, it is important to note that the DNP measurements are more sensitive to dendritic structures, as they are performed at higher magnetic field and microwave frequencies, resulting in skin depth on the scale of 100s of nm.

Finally, to further understand how current density influences lithium morphology— including particle size, shape, and surface roughness—we employed SEM. While DNP and EPR provided strong evidence for morphological differences, visual confirmation is essential to corroborate our findings. A major challenge in SEM analysis is that the metallic deposits are embedded deep within the polymer matrix. To address this, the polymer was gently stretched before imaging to expose both surface-level and partially embedded metal particles. Only particles visible within the imaging window (some exposed, some still embedded) were used for particle size analysis. It is important to highlight that the cycled polymer samples were carefully disassembled to avoid any residual fragments sticking to the polymer from the lithium metal electrodes. To verify this, EPR measurements were performed, confirming the absence of bulk Li metal and ensuring that the analysis was focused solely on lithium microstructures.

SEM images were acquired from multiple regions for samples prepared with both high (0.5 mA/cm^2^) and low (0.025 mA/cm^2^) current density (Figures S6 and S7). The results revealed a clear difference in morphology and particle size distribution. Lithium deposits formed under high current density predominantly consisted of smaller Li nanostructures, while those formed under low current density exhibited a broader size range, including large, micron-sized smooth chunks. This trend directly supports the observations made by EPR, where broader distributions suggested more heterogeneous morphologies.

To quantify the particle sizes, we used a grayscale profile analysis method. A line was drawn across each particle in the SEM image, and the fwhm of the grayscale intensity profile was used to estimate the particle size (Figure S8). This approach minimized bias and errors that can arise from manually selecting sizes, especially given the heterogeneity and random nature of the particles. Figure 4a presents violin plots of the particle size distribution. Based on the analysis of about 150–200 particles per condition, the distributions were clearly distinct: high current density samples showed a narrow distribution of smaller particles, while low current density samples showed a wider distribution spanning from hundreds of nanometers to several micrometers. Importantly, particle size alone does not capture the complete morphological differences. For example, some large structures were observed even in high current density samples; however, their surfaces were significantly rougher and nanostructured compared to similarly sized particles in the low current density condition, which appeared much smoother. This difference likely reflects more uniform lithium plating and stripping at lower current densities. Representative SEM images from different regions in samples cycled at each condition (Figures 4b,c and S9) clearly highlight these morphological differences.

**Figure 4:**
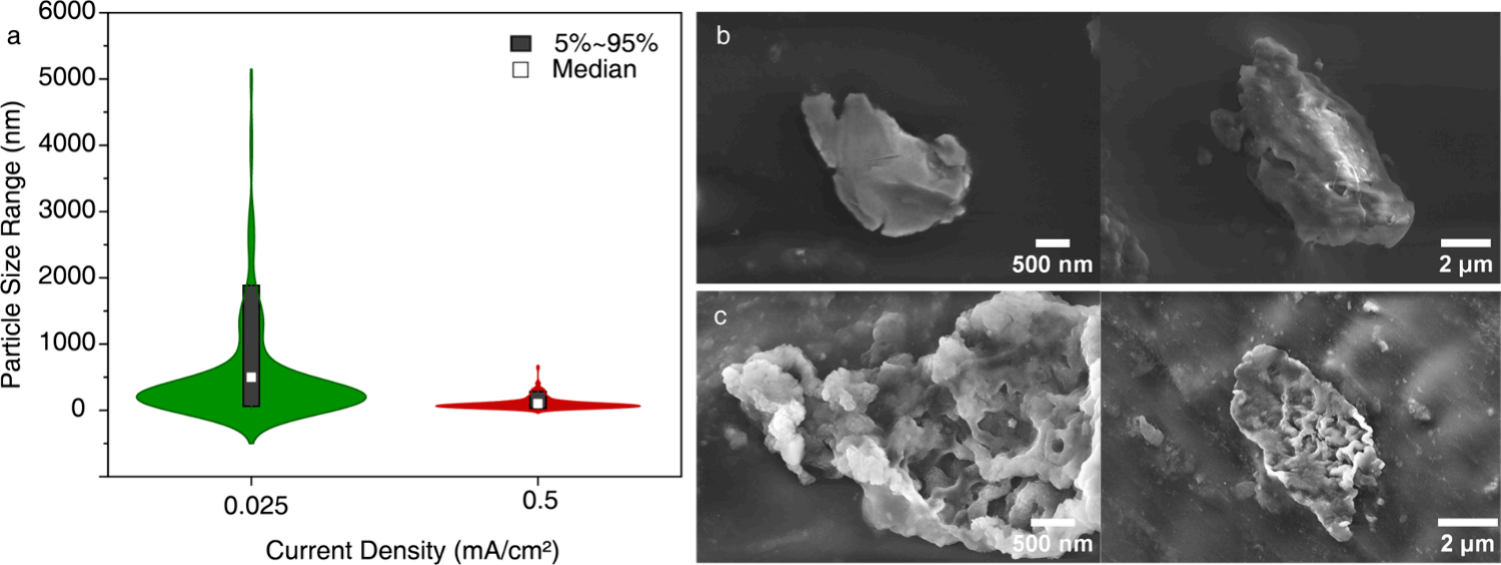
(a) Violin plot showing the particle size distribution of lithium deposits obtained from SEM analysis for samples cycled at 0.025 and 0.5 mA/cm^2^. The gray bars indicate the range from the 5th to 95th percentile and the median. SEM images of the lithium deposits formed under (b) low and (c) high current densities.

To conclude, our study presents a systematic approach to characterize morphological differences in lithium deposition under different cycling conditions. We demonstrated the strength of DNP-enhanced NMR in distinguishing these variations through differences in the enhancement factors and the shape of the metallic Li resonance. Deposits formed under high current density, which are smaller in size and lead to faster cell failure, exhibited significantly higher DNP enhancement factors compared with the larger structures formed under low current density. As a result, DNP is uniquely sensitive to nanoscale (and often needle-like) lithium dendrites, which pose the greatest risk to battery lifetime (in general) and safety (in flammable liquid electrolytes). Their small size and high reactivity make them particularly challenging to detect by conventional techniques. DNP overcomes this limitation, providing not only morphological contrast but also access to buried SEI chemistry. Complementary EPR analysis further revealed that dendrites with different sizes and morphologies possess distinct spectral characteristics and microwave saturation behaviors, indicative of longer electron relaxation times in dendritic structures. Electron microscopy provided direct visual confirmation, highlighting clear differences in the particle size distribution and surface roughness.

Overall, this study not only introduces a robust strategy for characterizing lithium morphology but also establishes a powerful, multimodal framework for identifying formation of dendritic structures in energy storage materials. These insights pave the way for future efforts aimed at understanding and mitigating dendrite formation in Li-metal batteries and beyond.

## Supplementary Material





## Notes

Methods, micro-CT analysis, OE-DNP enhancement vs current density, chemical exchange saturation transfer (CEST) experiment, SEI analysis, EPR properties of Li metal, SEM analysis and skin depth calculations (PDF)
3D micro-CT reconstruction of the cross-section of a cell cycled in 0.5 mA/cm^2^, related to Figure S1 (MP4)
