# Supplementary material for: Sensitive Detection of Dendritic Lithium Morphologies by Dynamic Nuclear Polarization
Source: J Phys Chem Lett. 2025 Aug 20;16(34):8792–8. doi: 10.1021/acs.jpclett.5c02140 (PMC12400404; doi:10.1021/acs.jpclett.5c02140)
Supplement: Supplementary file 1 [file jz5c02140_si_001.pdf]

Supporting Information

For

# Sensitive Detection of Dendritic Lithium Morphologies by Dynamic Nuclear Polarization

Nadav Maimon#(1), Ayan Maity#(1), Xiao-Meng Sui(2), Michal Leskes(1)*

(1)Department of Molecular Chemistry and Materials Science, Weizmann Institute of Science, Rehovot, Israel, 761000

(2) Department of Chemical Research Support, Weizmann Institute of Science, Rehovot, Israel, 761000

# equal contribution

*michal.leskes@weizmann.ac.il

## Contents

Contents ........ 1

1. Methods........ 2

  1.1. Preparation of Polymer Electrolyte........ 2

  1.2. Battery Assembly & Electrochemical Measurements........ 2

  1.3. Micro CT........ 2

  1.4. Sample Preparation for MAS-DNP, EPR and SEM Analysis........ 3

  1.5. DNP-NMR Measurements........ 3

  1.6. EPR Measurements........ 4

  1.7. SEM Analysis........ 4

2. Micro-CT analysis ........ 5

3. OE-DNP enhancement vs. Current density........ 5

4. Skin depth calculation........ 6

5. Chemical Exchange Saturation Transfer Experiments ........ 7

6. SEI analysis........ 8

7. EPR properties of Li metal ........ 8

8. SEM analysis ........ 9

References........11

## 1. Methods

### 1.1. Preparation of Polymer Electrolyte

The polymer electrolyte was prepared by mixing polyethylene oxide (PEO, MW = 600,000 g/mol) and lithium bis(trifluoromethanesulfonyl)imide (LiTFSI) in acetonitrile inside an argon-filled glove box. The molar ratio of ethylene oxide (EO) units to $Li^+$ ions was maintained at 18:1. The mixture was stirred continuously for at least 6–8 hours at 298 K until a uniform viscous solution was obtained. This solution was then cast into a circular Teflon mold and dried under a nitrogen flow at 80 °C, followed by vacuum drying for a minimum of 12 hours. The resulting polymer electrolyte film was then ready for use.

### 1.2. Battery Assembly & Electrochemical Measurements

Symmetric cells (2032-coin cells) were assembled using two lithium metal electrodes (thickness: 300–400 μm; diameter: 11 mm) and the prepared PEO–LiTFSI polymer electrolyte. The assembled cells were pre-heated at 80 °C for 3 hours to enhance interfacial contact between the Li metal and the polymer electrolyte. After heating, the cells were allowed to rest for 1 hour at 303 K before electrochemical testing. Cycling was performed using a Neware BTS-4000 Series battery tester at a controlled temperature of 303 K, under two different current densities: 0.025 $mA/cm^2$ and 0.5 $mA/cm^2$. For NMR studies, Li metal electrodes were made of 95% $^6$Li rich lithium.

### 1.3. Micro CT

To limit the exposure to humid air, in a glove box, samples were sliced into 2 mm wide stripes and inserted into a Kapton polyimide tube (5 mm in diameter), and then both ends of the tube were sealed with Parafilm. The 3D structure of the sample was obtained in two resolution steps, using Zeiss Xradia Versa 520 (Zeiss X-Ray Microscopy, USA) at 40 kV and 75 μA. First, a relatively large field of view (2.3 mm) scan with a pixel size of 2.3 μm, a 4X objective was used to collect 1600 projections over 360 °. After selecting a region of interest from the lower resolution scan, a high-resolution scan was performed. A total of 2400 projections with an exposure time of 5 s were taken over 360°. Final three-dimensional (3D) images were obtained with an isotropic voxel size of 0.8 μm. No source filter was used. Further image analysis was performed using the Feldkamp algorithm for filtered back projection. Image visualization was performed on Amira Software (Thermo Fisher Scientific, USA).

### 1.4. Sample Preparation for MAS-DNP, EPR and SEM Analysis

The cycled polymer electrolyte was carefully removed from the coin cells inside an argon-filled glove box. During disassembly, it was critical to ensure that no visible lithium metal fragments remained on the polymer, as their presence could interfere with subsequent analyses. The extracted polymer was then cut into smaller pieces for characterization. To ensure that no bulk Li metal remains on the polymer after removal, we rely on more than just visual inspection. MAS-DNP is highly sensitive to bulk lithium metal as its presence would strongly disrupt stable spinning due to its conductivity properties, which would be immediately evident during sample loading or acquisition. EPR is also highly sensitive to Li metal size; the presence of bulk lithium would result in large a/b ratio and broad FWHM, particularly at high microwave power. For this reason, all samples were measured at varying microwave power.

For DNP-NMR studies, the polymer pieces were packed into a 3.2 mm sapphire rotor closed with a tight Teflon insert and sealed with zirconia cap.

For EPR measurements, the polymer was loaded into an X-band quartz capillary. To avoid air exposure, the capillary was sealed using a custom-designed Teflon cap and vacuum grease.

For SEM imaging, the polymer was slightly stretched to expose embedded dendrites and then cut into small pieces. These were mounted onto carbon tape on an SEM stub. The sample holder was transported in double-sealed argon-filled bags. At the SEM facility, the sample was quickly transferred into the chamber with minimal air exposure (limited to approximately 5 seconds).

All measurements were performed immediately after cell disassembly to minimize dendrite decomposition or sample alteration. The samples were all measured in less than one hour, ensuring that each was freshly prepared and characterized directly after electrochemical cycling.

### 1.5. DNP-NMR Measurements

DNP-NMR measurements were performed using a Bruker Avance Neo spectrometer operating at 9.4 T, equipped with a 263 GHz gyrotron and an integrated sweep coil. The experiments employed 3.2 mm low-temperature DR DNP probe. Sample temperatures during the experiments were maintained around 98 K without microwaves and approximately 109 K when microwave irradiation was applied. All spectral analyses were carried out using Bruker TopSpin software, and DMFit was employed for spectral fitting and simulation[1].

### 1.6. EPR Measurements

Continuous-wave (CW) EPR measurements in the X-band (~9.4 GHz) were conducted using a Bruker Magnettech ESR5000 spectrometer, employing a modulation frequency of 100 kHz. Spectra were acquired at two different temperatures: room temperature (298 K) and cryogenic conditions (100 K).

### 1.7. SEM Analysis

SEM was conducted using a Zeiss Sigma field-emission SEM (FESEM). Imaging was performed with an accelerating voltage of 3–5 kV and a working distance maintained between 3–4 mm.

## 2. Micro-CT analysis

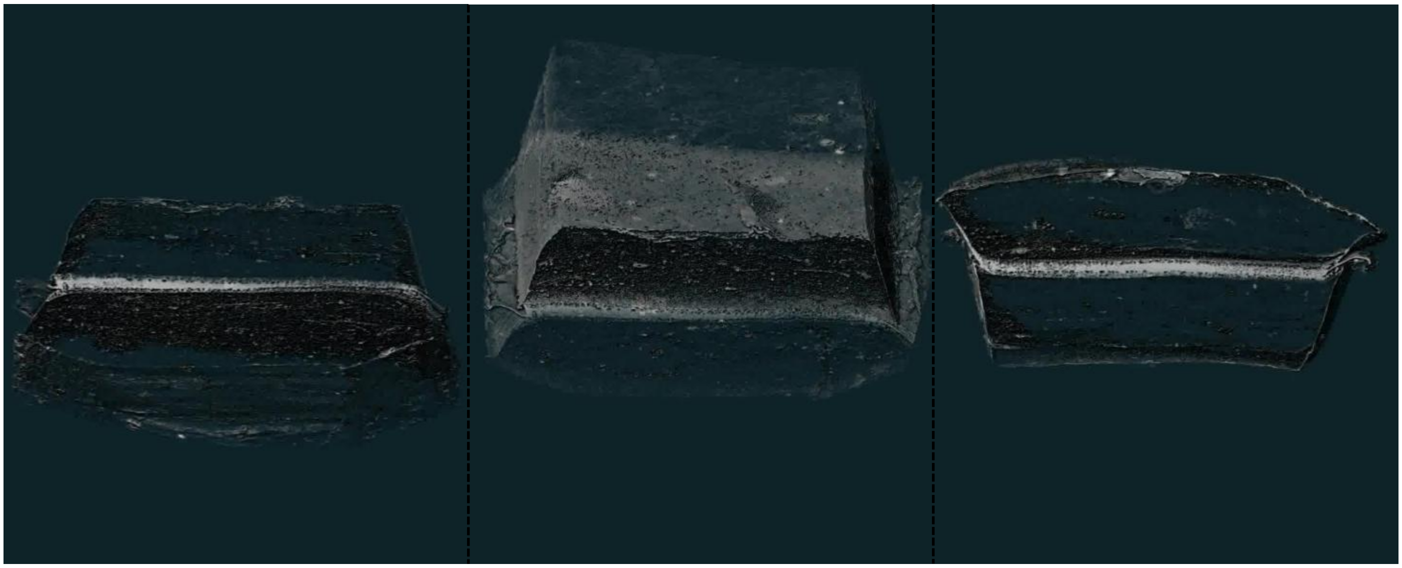

*Figure S1* 3D micro-CT reconstruction of the cross-section of a cell cycled in 0.5 mA/cm$^2$. The cell consists of two lithium metal electrodes and a polymer electrolyte (grey color), with embedded lithium dendrites visible as black dots within the polymer matrix. Further information is provided in the supplementary video.

## 3. OE-DNP enhancement vs. Current density

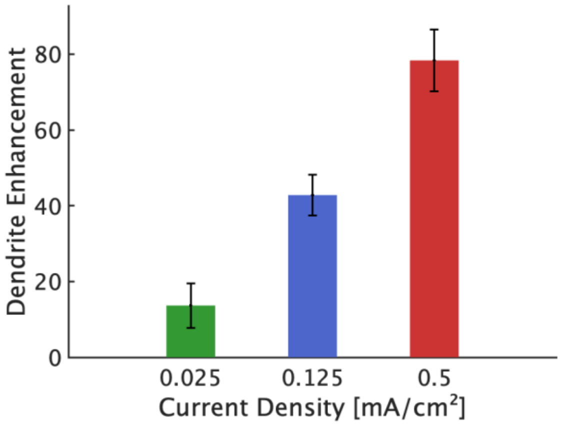


*Figure S2* Dendrite enhancement vs. current density for samples cycled at 0.025 mA/cm$^2$ (green), 0.125 mA/cm$^2$ (blue), and 0.5 mA/cm$^2$ (red). Error bars represent an average of three samples.

## 4. Skin depth calculation

The skin depth of microwave excitation is given by:

$$d = \left(\frac{\rho}{\pi \mu_0 \mu_r \upsilon}\right)^{\frac{1}{2}}$$

Where: $d$-skin depth, $\rho$ -Li metal resistivity[2], $\mu_0$- permeability of free space, $\mu_r$-relative permeability of lithium metal[3], $\upsilon$-microwave frequency. For 263 GHz:

$$d_{100K} = \left(\frac{1.73 * 10^{-8}\ [kg * m^2 * s^{-3} * A^{-2}]}{4 * \pi^2 * 10^{-7}\ [kg * m * s^{-2} * A^{-2}] * 1.0000142 * 263\ * 10^9\ [s^{-1}]}\right)^{\frac{1}{2}} = 129\ [nm]$$

$$d_{RT} = \left(\frac{9.47 * 10^{-8}\ [kg * m^2 * s^{-3} * A^{-2}]}{4 * \pi^2 * 10^{-7}\ [kg * m * s^{-2} * A^{-2}] * 1.0000142 * 263\ * 10^9\ [s^{-1}]}\right)^{\frac{1}{2}} = 302\ [nm]$$

For X-band (9.465 GHz):

$$d_{RT} = \left(\frac{9.47 * 10^{-8}\ [kg * m^2 * s^{-3} * A^{-2}]}{4 * \pi^2 * 10^{-7}\ [kg * m * s^{-2} * A^{-2}] * 1.0000142 * 9.465\ * 10^9\ [s^{-1}]}\right)^{\frac{1}{2}} = 1.59\ [\mu m]$$

## 5. Chemical Exchange Saturation Transfer Experiments

Chemical exchange saturation transfer (CEST) is used to probe exchange rates between two chemical environments. In this study, we investigate the exchange between lithium at the metal-SEI interface[4]. During DNP-enhanced NMR, both metallic and SEI signals are enhanced. A selective saturation pulse is applied at the metallic resonance, followed by a delay period, after which the SEI signal is monitored. If the SEI peak intensity decreases, it indicates that saturation has been transferred from the dendrites to the SEI. We have previously shown that this process of saturation transfer is due to charge transfer. To account for heating effects, each experiment is compared to a control with an off-resonance saturation pulse. The CEST effect is calculated as the ratio of the SEI signal integral with and without on-resonance saturation, normalized by the saturation efficiency at the dendrite resonance. **Figure S3a** compares the CEST effect for needle-like dendrites formed at 0.5 mA/cm$^2$ and mossy lithium at 0.025 mA/cm$^2$. As expected, the needle-like dendrites exhibit a larger CEST effect, consistent with their higher surface area and greater metal-SEI contact. These results support the conclusion that the SEI enhancement arises from polarization transfer via lithium exchange[5].

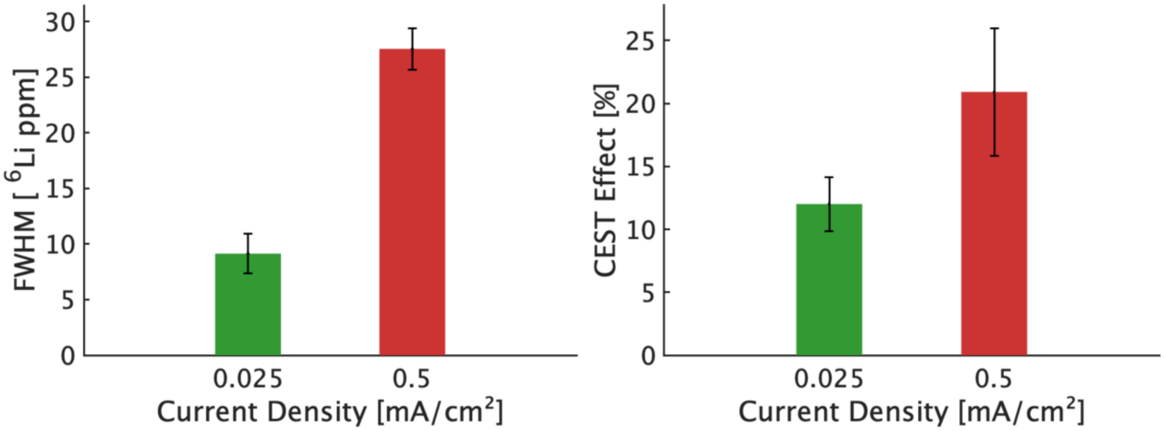


***Figure S3*** *(a) FWHM of the dendrite peak vs. current density for samples cycled at 0.025 mA/cm$^2$ (green) and 0.5 mA/cm$^2$ (red). (b) CEST effect vs. current density for the same conditions, acquired at 100K with a 60s recycle delay and 8 kHz MAS. The saturation pulse length was 300ms with 500 Hz RF amplitude. Error bars represent an average of three samples.*

## 6. SEI analysis

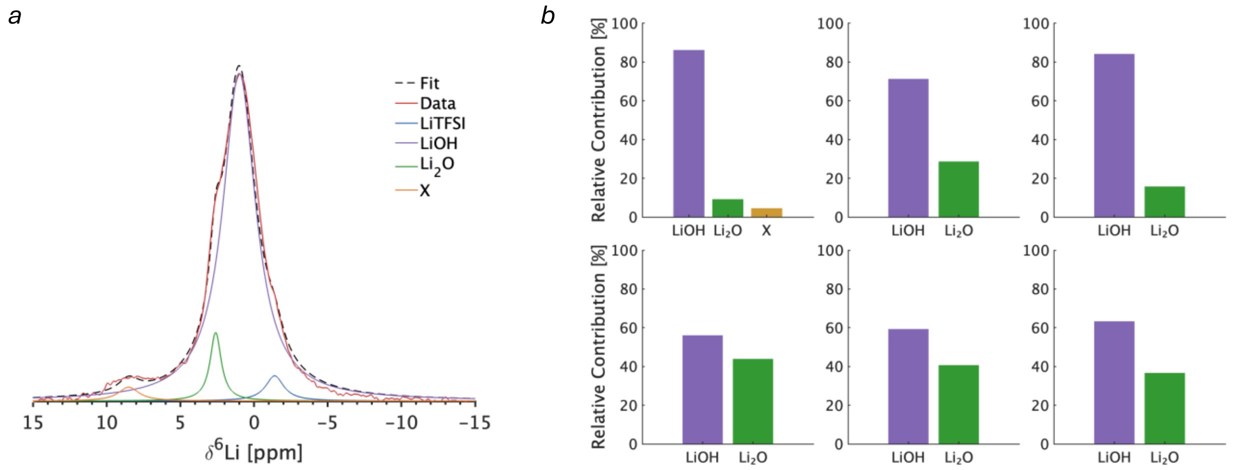


***Figure S4*** *(a) Example of SEI peak decomposition for a cell cycled at 0.5 mA/cm$^2$: experimental data (red), overall fit (dashed black), LiTFSI salt in the polymer (blue), LiOH (purple), Li$_2$O (green), and an unidentified component marked X (yellow). (b) SEI composition from peak decomposition for three cells cycled at 0.5 mA/cm$^2$ (top) and 0.025 mA/cm$^2$ (bottom).*

## 7. EPR properties of Li metal

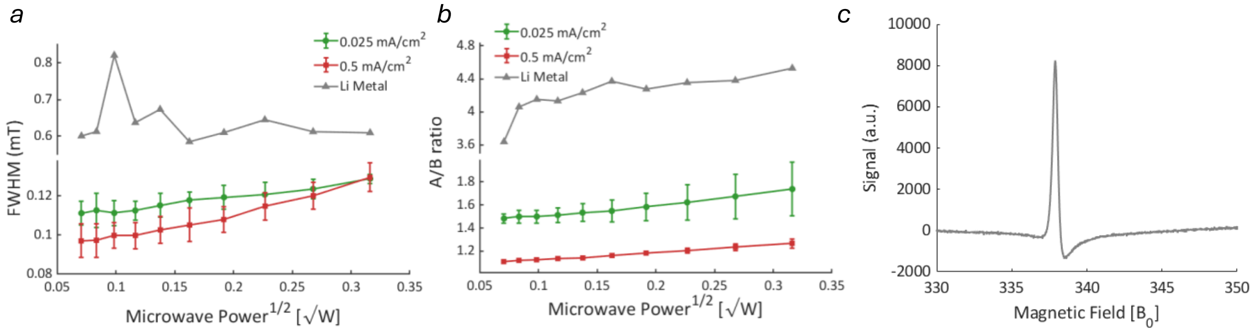


***Figure S5*** *A/B ratio (a) and FWHM (b) of EPR line vs. squared microwave power for dendrites cycled with 0.025 mA/cm$^2$ (green), 0.5 mA/cm$^2$ (red), and a lithium metal bulk (grey). Error bars represent an average of three samples. (c) EPR line shape of a bulk lithium metal measured with 100 mW microwave power.*

## 8. SEM analysis

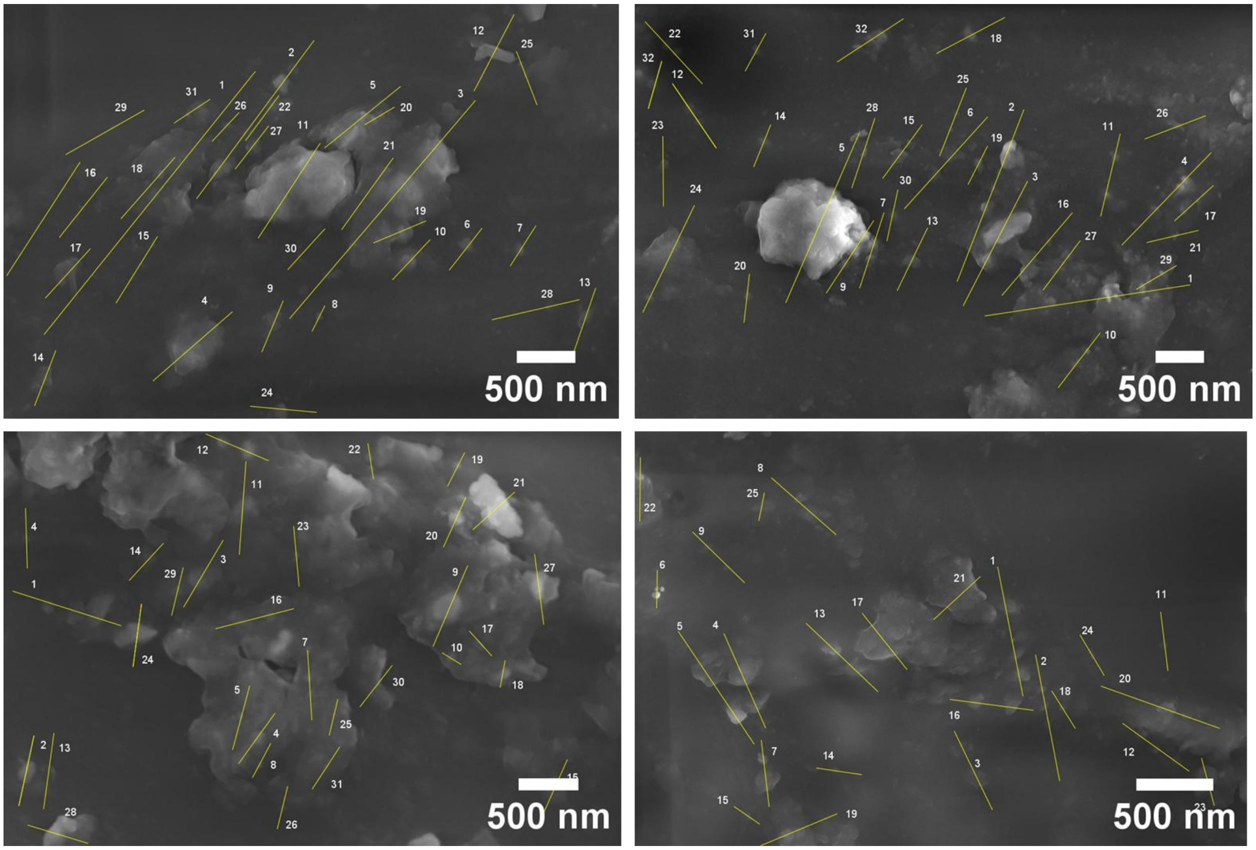


***Figure S6*** *SEM images of lithium dendrites embedded within the polymer electrolyte, collected from different locations and batches under the 0.5 mA/cm$^2$ current density cycling condition. The lines are used for the generation of Grayscale line profiles of various dendrites, and the corresponding full width at half maximum (FWHM) values were used to estimate particle sizes.*

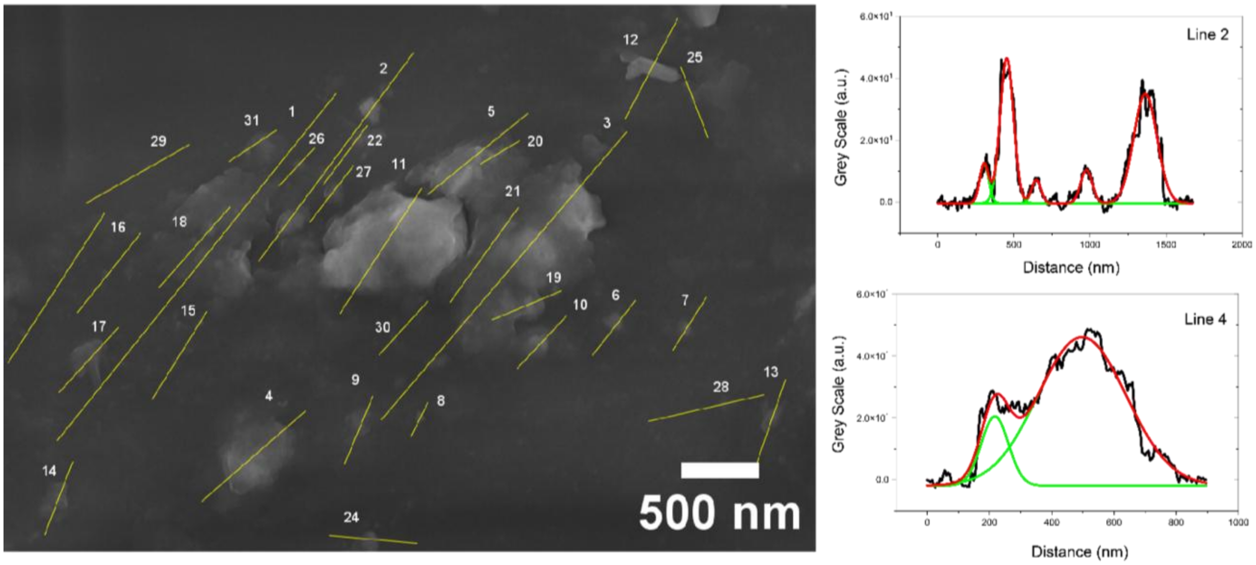


***Figure S7*** *SEM image showing lithium dendrites embedded within the polymer electrolyte under the 0.5 mA/cm*$^2$ *current density cycling condition (right). Grayscale line profiles were drawn across selected dendrites (left), and the FWHM values were extracted from these profiles to estimate particle sizes.*

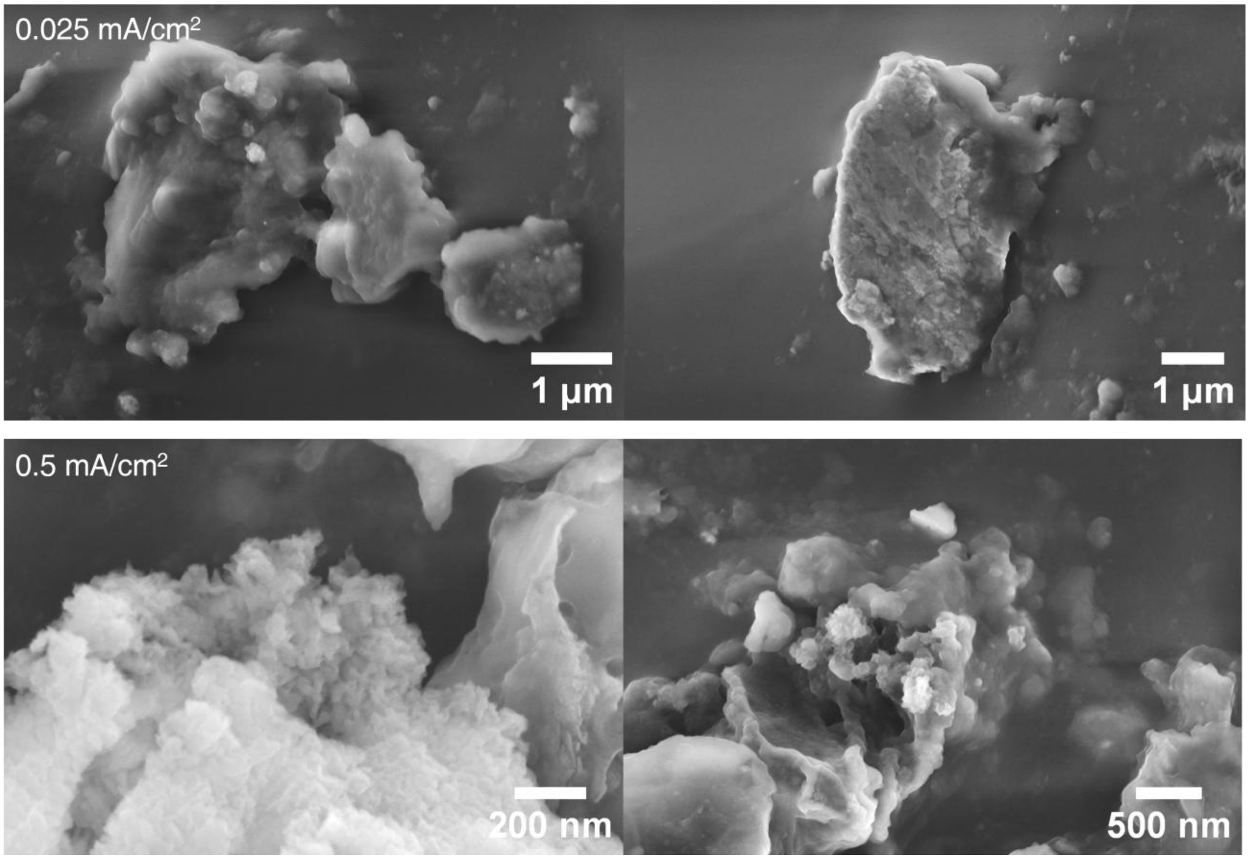


***Figure S9*** *Additional SEM images of the dendrites formed under low and high current densities, respectively. Dendrites generated at 0.5 mA/cm2 exhibit significantly greater surface roughness and a more nanostructured appearance, in contrast to the smoother, more compact structures observed at 0.025 mA/cm*$^2$*.*

## References


(1) Massiot, D.; Fayon, F.; Capron, M.; King, I.; Le Calvé, S.; Alonso, B.; Durand, J. O.; Bujoli, B.; Gan, Z.; Hoatson, G. Modelling One- and Two-Dimensional Solid-State NMR Spectra. *Magnetic Resonance in Chemistry* **2002**, *40* (1), 70–76. https://doi.org/10.1002/mrc.984.

(2) *CRC Handbook of Chemistry and Physics*, 97th, section 12-42 ed.; **2016**. https://doi.org/10.1201/9781315380476.

(3) Zheng, B.; Liu, X.; Xiang, Y. Solid-State Nuclear Magnetic Resonance Studies of Lithium and Sodium Metal Batteries. *The Journal of Physical Chemistry C* **2024**, *128* (44), 18659–18677. https://doi.org/10.1021/acs.jpcc.4c05822.

(4) Columbus, D.; Arunachalam, V.; Glang, F.; Avram, L.; Haber, S.; Zohar, A.; Zaiss, M.; Leskes, M. Direct Detection of Lithium Exchange across the Solid Electrolyte Interphase by $^{7}$Li Chemical Exchange Saturation Transfer. *J Am Chem Soc* **2022**, *144* (22), 9836–9844. https://doi.org/10.1021/jacs.2c02494.

(5) Maity, A.; Svirinovsky-Arbeli, A.; Buganim, Y.; Oppenheim, C.; Leskes, M. Tracking Dendrites and Solid Electrolyte Interphase Formation with Dynamic Nuclear Polarization—NMR Spectroscopy. *Nat Commun* **2024**, *15* (1), 9956. https://doi.org/10.1038/s41467-024-54315-w.
